# Family- and population-based designs identify different rare causal variants

**DOI:** 10.1186/1753-6561-5-S9-S36

**Published:** 2011-11-29

**Authors:** Xue Zhang, Hua He, Lili Ding, Tesfaye M Baye, Brad G Kurowski, Lisa J Martin

**Affiliations:** 1 Divisions of Biostatistics and Epidemiology, Cincinnati Children’s Hospital Medical Center, 3333 Burnet Avenue, Cincinnati, OH 45229, USA; 2 Asthma Research, Cincinnati Children’s Hospital Medical Center, 3333 Burnet Avenue, Cincinnati, OH 45229, USA; 3 Department of Pediatrics, University of Cincinnati School of Medicine, 231 Albert Sabin Way, Cincinnati, OH 45267, USA; 4 Physical Medicine and Rehabilitation, Cincinnati Children’s Hospital Medical Center, 3333 Burnet Avenue, Cincinnati, OH 45229, USA; 5 Human Genetics, Cincinnati Children’s Hospital Medical Center, 3333 Burnet Avenue, Cincinnati, Ohio 45229, USA

## Abstract

Both family- and population-based samples are used to identify genetic variants associated with phenotypes. Each strategy has demonstrated advantages, but their ability to identify rare variants and genes containing rare variants is unclear. To compare these two study designs in the identification of rare causal variants, we applied various methods to the population- and family-based data simulated by the Genetic Analysis Workshop 17 with knowledge of the simulated model. Our results suggest that different variants can be identified by different study designs. Family-based and population-based study designs can be complementary in the identification of rare causal variants and should be considered in future studies.

## Background

Missing heritability is a major challenge in the discovery of genetic variants responsible for complex disease [1]. One possible reason for the missing heritability is that the current genome approaches focus on common rather than rare variation [2]. However, it is increasingly recognized that rare variants may be responsible for complex disease etiology [3,4]. Thus the next generation of gene discovery should focus on identification of rare variants.

Both family-based and population-based samples have been used to identify variants associated with phenotypes. In recent years, population-based association studies have gained favor because increased power may be obtained [5,6]. On the other hand, family-based approaches, such as linkage, are optimally positioned to identify rare variants with large effects [7,8]. Because each type of design has strengths and limitations, studies have been conducted using both designs simultaneously. Successes have been reported for common variants, in which the same variants were detected by both designs [9,10]. However, inconsistency was also observed [11,12].

It is not clear how family- and population-based analyses behave on rare variants. By applying various methods to the population- and family-based data simulated by Genetic Analysis Workshop 17 (GAW17), we compared the power of different designs in the identification of rare (minor allele frequency [MAF] < 0.01) causal variants.

## Methods

Two data sets were analyzed [13]. One consists of 697 unrelated individuals; the other consists of 697 individuals from 8 extended families. Simulated Q1 phenotypes were used. Analyses were adjusted for age, sex, smoking status, and population stratification using principal components analysis.

### Gene-level analysis

We analyzed the family-based data with a two-point linkage analysis using Sequential Oligogenic Linkage Analysis Routines (SOLAR) 4.10, with identity-by-descent (IBD) matrices from fully informative markers provided by GAW17.

For the population-based data, we collapsed single-nucleotide polymorphism (SNP) information on each of the nine Q1 related genes using three methods. The first method was indicator coding, in which genetic information of a particular gene was dichotomized according to the presence or absence of at least one rare nonsynonymous variant. The second method was percent coding, in which genetic information of a particular gene was calculated as *S_i_* = *r_i_*/*n_i_*, where *n_i_* denotes the number of rare variants successfully genotyped for subject *i* and *r_i_* denotes the number of these variants that carry at least one copy of the minor allele. The third method was weighted-sum collapsing. This method assumes an additive allelic effect by recoding the genotypes into 0, 1, or 2 based on the copy number of the minor allele. Then the single-SNP effect for each of the rare nonsynonymous SNPs was examined using univariate regression. For SNPs with significant (*α* ≤ 0.1) negative effect, the genotypes were converted to 2, 1, and 0 from 0, 1, and 2, respectively. The genetic information of one particular gene was then summarized as the sum of the numeric genotypes of all rare nonsynonymous SNPs on the gene. The gene-Q1 association was then tested using linear regression.

### SNP-level analysis

In the population-based data, single-SNP association with Q1 was tested using linear regression. In the family-based data, single-SNP association was assessed using a measured genotype approach that compared polygenic models with or without each of the SNPs as a covariate [14]. The quantitative transmission disequilibrium test (QTDT) was performed using JMP Genomics 4.

Analyses were performed with knowledge of the simulated model.

## Results

### Gene-level analysis

In the family-based data, linkage (LOD ≥ 1) to Q1 phenotype was detected in all Q1-related genes, suggestive linkage (LOD ≥ 2) was detected in five genes, and strong linkage (LOD ≥ 3) was detected in only the *VEGFA* and *VEGFC* genes (Table 1).

**Table 1:** Two-point linkage analysis using family-based data

Gene	SNP with highest *β*		SNP with highest MAF		Number of simulation			
	
	SNP	*β*	SNP	MAF	LOD ≥ 1 (*α* = 10^−2^)	LOD ≥ 2 (*α* = 10^−3^)	LOD ≥ 3 (*α* = 10^−4^)	LOD ≥ 4 (*α* = 10^−5^)
*ARNT*	C1S6561	0.65721	C1S6533	0.011478	1	0	0	0
*ELAVL4*	C1S3181	0.76911	C1S3181	0.000717	3	0	0	0
			C1S3182	0.000717				
*FLT1*	C13S479	0.75946	C13S523	0.066714	4	0	0	0
*FLT4*	C5S5156	0.43010	C5S5133	0.001435	10	1	0	0
*HIF1A*	C14S1729	0.28532	C14S1734	0.012195	1	0	0	0
*HIF3A*	C19S4831	0.29287	C19S4799	0.000717	43	3	0	0
			C19S4815	0.000717				
			C19S4831	0.000717				
*KDR*	C4S1877	1.07706	C4S1878	0.164993	48	9	0	0
*VEGFA*	C6S2981	1.20645	C6S2981	0.002152	198	172	110	55
*VEGFC*	C4S4935	1.35726	C4S4935	0.000717	197	167	126	76

*α* values associated with LOD scores are calculated as described by Ott [15].

For the 200 simulated population-based data, genes *FLT1* and *KDR* showed high power in all three SNP collapsing methods, followed by the *VEGFC* and *VEGFA* genes. Power to detect the *ARNT*, *ELAVL4*, *HIF1A*, *FLT4*, and *HIF3A* genes was low (Table 2).

**Table 2:** Gene-phenotype association analysis using population-based data

Gene	Number of SNPs	Number of causal SNPs	Causal/total SNPs (%)	Number of simulations with gene detected at *α* = 0.01			Number of simulations with gene detected at *α* = 0.0001		
	
				Binary	Percent	Sum test	Binary	Percent	Sum test
*ARNT*	8	4	50	2	2	3	0	0	0
*ELAVL4*	3	2	67	2	2	2	0	0	0
*FLT1*	17	8	47	111	111	115	19	19	23
*FLT4*	5	2	40	0	0	5	0	0	0
*HIF1A*	5	3	60	3	3	2	0	0	0
*HIF3A*	6	3	50	0	0	1	0	0	0
*KDR*	9	8	89	98	162	151	17	52	55
*VEGFA*	2	1	50	16	16	20	1	1	1
*VEGFC*	1	1	100	86	86	86	11	11	11

Only rare (MAF < 0.01) nonsynonymous SNPs were used in the analyses.

### SNP-level analysis

We tested single-SNP association for the 39 causal SNPs in both the population-based and the family-based data. The highest power was observed for markers C13S522 and C13S523 in the population-based data and for markers C6S2981 and C4S4935 in the family-based data (Additional file 1). Although C6S2981 and C4S4935 are rare in the population, they are enriched in families. For SNPs with similar MAFs in both the population-based and the family-based data (C1S3181, C13S431, C4S1861, C4S1878, and C4S1884), our results showed similar power of identification.

Using the QTDT on family-based data, we also tested SNP association for SNPs on chromosomes 1, 4, 6, and 13. The QTDT showed overall lower power than the measured genotype approach (Additional file 1).

Because single-SNP association and the QTDT are two commonly used analysis methods for population- and family-based data, respectively, we compared the power of SNP identification of these two methods. Among the 32 rare causal SNPs in the population-based data, 3 were identified with greater than 50% power. Among the 11 rare causal SNPs that showed polymorphism in the family-based data, no true causal SNP was identified with greater than 50% power. In both analyses, high power was observed in common SNPs.

## Discussion and conclusions

Using data simulated by GAW17, in the current study we compared population-based and family-based designs for their ability to identify rare causal variants, as well as gene-level association. We found that the population-based and family-based designs can result in the identification of different causal variants and genes. Because the same underlying simulated model was used for both the family- and population-based data sets, these results suggest that both of these designs have roles in the discovery of rare variant association.

By comparing the identified and unidentified causal genes (Tables 1 and 2), we found several interesting characteristics. Both population- and family-based analysis identified particular genes most of the time (*KDR* and *FLT1* by population-based data; *VEGFA* and *VEGFC* by family-based data). In the family-based data, both *KDR* and *FLT1* have five polymorphic causal variants, whereas *VEGFA* and *VEGFC* included only a single causal variant each. Based on the expected performance of linkage, one might expect linkage to work better in genes with multiple variants. However, *VEGFA* and *VEGFC* show larger effects (*β* = 1.21 and 1.36, respectively); thus the ability to detect the *VEGF* gene may be more reflective of the effect than of the number of variants. On the other hand, the methods we used to identify gene-Q1 association in the population-based data rely largely on the probability to capture rare variants; thus a higher power for genes with more rare variants (*KDR* and *FLT1*) is not surprising.

When comparing SNP association and the measured genotype approach, we found that power is related to MAF (Additional file 1). When MAF is similar, these two methods show no difference. On the other hand, these two data sets identify different SNPs. Because similar approaches are used, this difference is likely due to the design. The results suggest that for SNPs that are rare in a population, a family-based design may provide an opportunity to enrich the rare SNPs, thus increasing the power to detect the SNP-phenotype association (e.g., C6S2981 and C4S4935). However, a family-based sample may lack polymorphism by chance. In this case, population sampling may be advantageous (e.g., C4S1877 and C4S1889).

When comparing linkage and association results from the family-based data (Table 1 and Additional file 1), we noticed that *FLT4* and *HIF3A* were identified by linkage, but the causal SNPs on these two genes were either nonpolymorphic or had no power to be identified even at the 0.01 level in the association test. Thus, when analyzing family-based data, linkage analysis may be advantageous in the identification of causal regions by using other genetic variations in the same region.

We also compared the association results at the SNP and gene levels from the population-based data (Table 2 and Additional file 1). It appears that gene-level association is not likely to be detected when SNP-level association is lacking. Collapsing the information of the rare SNPs on one particular gene may not enhance the power or provide additional information, as linkage analysis would.

Taken together, these results suggest that neither the family-based nor the population-based analysis we used is sufficient to identify causal variants of next-generation sequence-level data, especially in the context of rare variants. Given that the family-based design offers a variety of advantages (such as segregation with disease rather than just co-occurrence) that cannot be used for unrelated individuals and that may enrich rare variants, the family-based design may also be valuable for genome-wide SNP scanning for novel causal variants. Population- and family-based designs can be complementary and should both be considered in future genome-wide association studies.

## Competing interests

The authors declare that there are no competing interests.

## Authors’ contributions

XZ carried out the design of the study, analyses using the family-based data and drafted the manuscript. HH carried out analyses using the population-based data. LD, TMB and BGK participated in the discussion, and helped to edit the manuscript. LJM conceived of and oversaw the study. All authors read and approved the final manuscript.

## Supplementary Material

Additional file 1 **Power of the association test in population and family-based data** Power% is the number of replicates detected divided by the number of replicates analyzed multiplied by 100. The QTDT was performed with 100 replicates; other analyses were performed with 200 replicates. No result was generated by the QTDT for C1S3181 and C4S1890. SNPs with MAF > 0.01 are shaded. na, not applicable. Click here for file
