# Supplementary material for: Family- and population-based designs identify different rare causal variants
Source: BMC Proc. 2011 Nov 29;5(Suppl 9):S36. doi: 10.1186/1753-6561-5-S9-S36 (PMC3287872; doi:10.1186/1753-6561-5-S9-S36)
Supplement: Additional file 1 — Power of the association test in population and family-based data Power% is the number of replicates detected divided by the number of replicates analyzed multiplied by 100. The QTDT was performed with 100 replicates; other analyses were performed with 200 replicates. No result was generated by the QTDT for C1S3181 and C4S1890. SNPs with MAF > 0.01 are shaded. na, not applicable. [file 1753-6561-5-S9-S36-S1.pdf]

**Table 3 - Power of the association test in population and family-based data**

Power% is the number of replicates detected divided by the number of replicates analyzed multiplied by 100. The QTDT was performed with 100 replicates; other analyses were performed with 200 replicates. No result was generated by the QTDT for C1S3181 and C4S1890. SNPs with MAF > 0.01 are shaded.

na, not applicable.

| | | | Population-based data | | | Family-based data | | | | |
|---|---|---|---|---|---|---|---|---|---|---|
| | | | | Single-SNP association | | | Measured genotype approach | | QTDT | |
| Gene | SNP | $\beta$ | MAF | Power% at $\alpha$ = 0.01 | Power% at $\alpha$ = 0.0001 | MAF | Power% at $\alpha$ = 0.01 | Power% at $\alpha$ = 0.0001 | Power% at $\alpha$ = 0.01 | Power% at $\alpha$ = 0.0001 |
| *ARNT* | C1S6533 | 0.5619 | 0.011478 | 65 | 13 | 0.005 | 9 | 1 | 0 | 0 |
| | C1S6537 | 0.64454 | 0.000717 | 3 | 0 | 0 | na | na | na | na |
| | C1S6540 | 0.24129 | 0.001435 | 1 | 0 | 0.0029 | 1 | 0 | 0 | 0 |
| | C1S6542 | 0.46026 | 0.002152 | 9 | 0 | 0 | na | na | na | na |
| | C1S6561 | 0.65721 | 0.000717 | 1 | 0 | 0 | na | na | na | na |
| *ELAVL4* | C1S3181 | 0.76911 | 0.000717 | 5 | 0 | 0.0007 | 7 | 0 | – | – |
| | C1S3182 | 0.30432 | 0.000717 | 1 | 0 | 0 | na | na | na | na |
| *FLT1* | C13S320 | 0.19605 | 0.001435 | 2 | 0 | 0.0036 | 5 | 0 | 0 | 0 |
| | C13S399 | 0.39602 | 0.000717 | 1 | 0 | 0 | na | na | na | na |
| | C13S431 | 0.74136 | 0.017217 | 78 | 24 | 0.0187 | 93 | 50 | 50 | 5 |
| | C13S479 | 0.75946 | 0.000717 | 1 | 0 | 0 | na | na | na | na |
| | C13S505 | 0.4485 | 0.000717 | 1 | 0 | 0 | na | na | na | na |
| | C13S514 | 0.56643 | 0.000717 | 1 | 0 | 0.0022 | 4 | 0 | 0 | 0 |
| | C13S522 | 0.6183 | 0.027977 | 100 | 100 | 0.0093 | 74 | 18 | 30 | 3 |
| | C13S523 | 0.64997 | 0.066714 | 100 | 100 | 0.0194 | 97 | 60 | 44 | 8 |

| | | | | | | | | | | |
|---|---|---|---|---|---|---|---|---|---|---|
| | C13S524 | 0.62223 | 0.004304 | 99 | 69 | 0 | na | na | na | na |
| | C13S547 | 0.52601 | 0.000717 | 1 | 0 | 0 | na | na | na | na |
| | C13S567 | 0.17493 | 0.000717 | 1 | 0 | 0 | na | na | na | na |
| *FLT4* | C5S5133 | 0.15986 | 0.001435 | 7 | 0 | 0 | na | na | na | na |
| | C5S5156 | 0.4301 | 0.000717 | 1 | 0 | 0 | na | na | na | na |
| *HIF1A* | C14S1718 | 0.15382 | 0.000717 | 2 | 0 | 0 | na | na | na | na |
| | C14S1729 | 0.28532 | 0.002152 | 0 | 0 | 0 | na | na | na | na |
| | C14S1734 | 0.21203 | 0.012195 | 29 | 2 | 0.0007 | 2 | 0 | na | na |
| | C14S1736 | 0.21716 | 0.000717 | 1 | 0 | 0 | na | na | na | na |
| *HIF3A* | C19S4799 | 0.28351 | 0.000717 | 0 | 0 | 0 | na | na | na | na |
| | C19S4815 | 0.53114 | 0.000717 | 2 | 0 | 0 | na | na | na | na |
| | C19S4831 | 0.29287 | 0.000717 | 2 | 0 | 0.0022 | 0 | 0 | na | na |
| *KDR* | C4S1861 | 0.56311 | 0.002152 | 5 | 0 | 0.0022 | 2 | 0 | 1 | 0 |
| | C4S1873 | 0.58301 | 0.000717 | 4 | 0 | 0.0014 | 6 | 0 | 0 | 0 |
| | C4S1874 | 0.47262 | 0.000717 | 3 | 0 | 0 | na | na | na | na |
| | C4S1877 | 1.07706 | 0.000717 | 92 | 53 | 0 | na | na | na | na |
| | C4S1878 | 0.13573 | 0.164993 | 55 | 10 | 0.137 | 58 | 13 | 28 | 3 |
| | C4S1879 | 0.6183 | 0.000717 | 3 | 0 | 0 | na | na | na | na |
| | C4S1884 | 0.29558 | 0.020803 | 35 | 4 | 0.0194 | 40 | 3 | 8 | 1 |
| | C4S1887 | 0.29558 | 0.000717 | 3 | 0 | 0 | na | na | na | na |
| | C4S1889 | 0.94133 | 0.000717 | 92 | 53 | 0 | na | na | na | na |
| | C4S1890 | 0.42407 | 0.002152 | 1 | 0 | 0.0007 | 0 | 0 | – | – |
| *VEGFA* | C6S2981 | 1.20645 | 0.002152 | 30 | 3 | 0.033 | 100 | 100 | 100 | 100 |
| *VEGFC* | C4S4935 | 1.35726 | 0.000717 | 46 | 6 | 0.0222 | 100 | 100 | 100 | 100 |
